# Peripheral blood mRNA-based prognostic model for progression to metastatic castration-resistant prostate cancer and identification of SLC6A8 as a glycolysis-promoting biomarker

**DOI:** 10.3389/fonc.2026.1942820

**Published:** 2026-10-14

**Authors:** Sicong Li, Heling Zhou, Yi Wu, Yingming Xiao, Zeng Li, Shukui Zhou, Mingli Wu, Yan Wang, Hong Liao, Shengke Yang

**Affiliations:** 1 National Cancer Center/National Clinical Research Center for Cancer/Cancer Hospital, Chinese Academy of Medical Sciences and Peking Union Medical College, Beijing, China; 2 Department of Nursing, Sichuan Clinical Research Center for Cancer, Sichuan Cancer Hospital and Institute, Sichuan Cancer Center, University of Electronic Science and Technology of China, Chengdu, Sichuan, China; 3 Department of Urology, Sichuan Clinical Research Center for Cancer, Sichuan Cancer Hospital and Institute, Sichuan Cancer Center, Affiliated Cancer Hospital of University of Electronic Science and Technology of China, Chengdu, Sichuan, China; 4 Department of Surgery, Medical Center Hospital of Qionglai City, Qionglai, Sichuan, China

**Keywords:** glycolysis, metastatic castration-resistant prostate cancer, metastatic hormone-sensitive prostate cancer, prognostic model, single-cell RNA sequencing

## Abstract

**Objectives:**

Approximately 35% of patients with metastatic hormone-sensitive prostate cancer (mHSPC) progress to metastatic castration-resistant prostate cancer (mCRPC), highlighting the limitations of PSA as a stand-alone monitoring biomarker. This study aimed to construct a peripheral blood mRNA-based prognostic model for progression to mCRPC, evaluate the prognostic value of the identified biomarkers, and explore the biological role of the top-performing gene in prostate cancer cells.

**Methods:**

Single-cell transcriptomic data (GSE288878) were analyzed to screen for mCRPC-related differentially expressed genes (DEGs). A prospective cohort study was conducted on newly diagnosed mHSPC patients who received first-line androgen deprivation therapy (ADT)-based treatment, from whom peripheral blood samples were collected for transcriptome sequencing. The intersection of DEGs from GSE288878 and our cohort defined the gene signature. Following random splitting of the dataset into development and validation sets (7:3), we used an Elastic Net-regularized Cox model to derive a prognostic model for mCRPC-free survival. TCGA prostate cancer samples were used to analyze the association between biomarker genes and progression-free survival (PFS). *In vitro* experiments investigated the biological role of the gene with prognostic significance in blood and cancer tissues.

**Results:**

Five candidate genes (SLC25A39, GRB10, SELENBP1, SLC6A8, and YBX3) were identified as closely associated with mCRPC. We constructed a prognostic model, with an AUC for predicting 1-year mCRPC-free survival of 0.89 (0.80–0.97) in the development set and 0.83 (0.62–1.00) in the validation set. We identified elevated SLC6A8 expression in peripheral blood as an independent risk factor for progression to mCRPC. In the TCGA database, elevated SLC6A8 expression in prostate cancer tissue was identified as an independent risk factor for shorter PFS. Pharmacological inhibition of SLC6A8 significantly suppressed CRPC cell proliferation, decreased glycolytic metabolite levels, and downregulated glycolysis-associated gene expression.

**Conclusions:**

The prognostic model effectively predicted progression to mCRPC in patients with mHSPC receiving first-line therapy. SLC6A8 may serve as a prognostic biomarker and promote prostate cancer progression through enhanced glycolysis.

## 1 Introduction

Prostate cancer is one of the most commonly diagnosed malignancies among men worldwide. Furthermore, mortality attributable to this disease is anticipated to surge by 85%, climbing from 375,000 deaths in 2020 to roughly 700,000 by 2040 (1, 2). Although first-line treatment with ADT combined with androgen receptor pathway inhibitors (ARPIs) significantly improves overall survival (OS) and PFS in patients with mHSPC, approximately 35% of newly diagnosed mHSPC patients still progress to mCRPC within 5 years (3, 4). Awais Paracha et al. conducted a cohort study and found that approximately 78% of mHSPC patients eventually progressed to mCRPC (5). Progression from mHSPC to mCRPC may be categorized as radiographic progression, biochemical progression, or combined biochemical and radiographic progression (6). Wenzel et al. identified through a cohort study that 23% of mHSPC patients were later diagnosed with mCRPC based solely on radiographic progression, and 35% based exclusively on biochemical progression (6). In a single-center retrospective registry of 229 individuals with mHSPC, multivariable modeling further indicated that visceral metastatic burden and early exposure to either docetaxel-based chemotherapy or androgen receptor axis-targeted agents independently predicted this discordant progression pattern (7). In another retrospective study, Tsutsumi et al. revealed that isolated radiographic progression occurred in 20.1% (34/169) of patients upon progression to CRPC. This imaging-defined phenotype demonstrated a markedly reduced median overall survival relative to progression characterized solely by PSA elevation (8). The above results indicate the necessity of regular imaging examinations even if PSA elevation does not occur during mHSPC treatment. Exploring biomarkers is expected to reduce clinical reliance on imaging examinations and achieve more accurate personalized monitoring. Using three GEO datasets, Shao et al. established a ferroptosis-related gene panel to differentiate HSPC from CRPC in tissue samples. Nevertheless, the invasive nature of tumor biopsy and inability to track molecular changes serially limit its application for dynamic disease monitoring compared with peripheral blood-based approaches (9). AR-V7, an androgen receptor splice variant, was detected in circulating tumor cells(CTCs) and was found to predict resistance to abiraterone/enzalutamide treatment (10, 11). The rarity of CTCs in peripheral blood, the technical challenge of efficiently and specifically isolating them from massive background cell populations, and the lack of standardized extraction methods across different platforms collectively restrict the widespread clinical application of CTC detection.

A prognostic model based on peripheral blood mRNA demonstrates unique value: first, non-invasive accessibility, requiring only routine venous blood collection while avoiding repeated biopsy or high-radiation imaging examinations, particularly suitable for middle-aged and elderly patients requiring long-term follow-up; second, dynamic monitoring capability, enabling real-time tracking of molecular evolution during disease progression to compensate for the “single time-point” limitation of tissue biomarkers and provide potential for early identification of the CRPC transformation window; third, technical feasibility, with abundant leukocyte content (~10^9^ cells/L), superior RNA stability compared to CTCs, and highly standardized extraction methods with good cross-platform reproducibility.

In this study, we first performed single-cell transcriptomic analysis of the GSE288878 dataset to identify genes closely associated with mCRPC. Subsequently, a prospective cohort study was conducted at Sichuan Cancer Hospital, in which 147 patients with mHSPC receiving first-line ADT-based therapy ultimately completed follow-up. Additionally, we evaluated the prognostic value of each model gene for PFS in the TCGA database. Finally, the potential biological roles of genes exhibiting prognostic significance were investigated in prostate cancer cells.

## 2 Materials and methods

### 2.1 Reagents and kits

Cell viability was assessed using the Cell Counting Kit-8 (CCK-8; catalog no. C0038, Beyotime, Beijing, China). Apoptosis was evaluated with the Annexin V-FITC/Propidium Iodide (PI) staining kit (catalog no. 556547, BD Biosciences, Franklin Lakes, NJ, USA). Total RNA was extracted using TRIzol Reagent (catalog no. 15596026, Thermo Fisher Scientific, Waltham, MA, USA). RNA sequencing libraries were prepared with the Illumina Stranded Total RNA Prep, Ligation kit (catalog no. 20040529, Illumina, San Diego, CA, USA). Protein detection was performed using enhanced chemiluminescence (ECL) substrate (catalog no. 32106, Thermo Fisher Scientific). The following primary antibodies were purchased from Proteintech (Wuhan, China): anti-HK2 (1:5000; catalog no. 22029-1-AP), anti-SLC6A8 (1:1000; catalog no. 20299-1-AP), anti-PFKP (1:5000; catalog no. 13389-1-AP), anti-ALDH1B1 (1:4000; catalog no. 85426-2-RR), and anti-β-Actin (1:20000; catalog no. 66009-1-Ig).

### 2.2 Single-cell data analysis

We obtained the scRNA-seq data of cells from GSM8777871 and GSM8777872 in the GSE288878 dataset from the Gene Expression Omnibus (GEO) database. GSM8777871 contained LNCaP_FGC cells, which were reported as HSPC cells, whereas GSM8777872 contained LNCaP_C4-2B cells, which were reported as CRPC cells (12, 13). To maintain data quality, we excluded cells with mitochondrial gene expression exceeding 10%, as well as those with gene expression levels below 200 or above 4500, and cells with RNA counts below 1000 or above 35000. Following the quality assessment of the cells, we applied the “NormalizeData” function to normalize the data. We computed the gene expression levels by taking the gene’s value, scaling it to a proportion, and multiplying by 10,000 for each sample. To avoid logarithmic calculations of zero, we added 1 before converting the values to natural logarithms. The “FindVariableFeatures” algorithm was then used to identify the top 2000 highly variable genes (HVGs). These HVGs were extracted, centered, and scaled using the “ScaleData” function before performing principal component analysis (PCA) on this selected gene subset. To align the entire dataset, the “anchor point” method was employed. This technique uses a small subset of data points from the same cell type present in two or more different single-cell sequencing datasets, integrating them to correct for batch effects (14). Seurat clusters cells into distinct groups based on their PCA scores, with each principal component (PC) serving as a “meta feature” that aggregates information from interrelated feature sets. The “RunPCA” function is used to conduct dimensionality reduction through PCA, focusing on the 2000 most variable genes previously identified. We first constructed cell clusters through Seurat’s spectral clustering framework, setting the resolution parameter to 1.2 in the “FindNeighbors” and “FindClusters” steps to obtain high-resolution groupings. The resulting clusters were then projected into a two-dimensional space using t-SNE for nonlinear dimensionality reduction. To identify cluster-specific markers, we applied the Wilcoxon rank-sum test via Seurat’s “FindAllMarkers” tool, retaining only those genes satisfying |logFC| ≥ 0.25, min.pct ≥ 0.25, and P < 0.05. We employed scMetabolism (v0.2.1) to quantify metabolic pathway activities across distinct cell subpopulations (15).

### 2.3 A prospective cohort study investigating peripheral blood genes related to mCRPC

This study was approved by the Ethics Committee for Medical Research and New Medical Technology of Sichuan Cancer Hospital (approval no. SCCHEC-02-2024-100) and was conducted in strict accordance with the Declaration of Helsinki. Written informed consent was obtained from all participants. We employed a prospective cohort study. We enrolled treatment-naïve mHSPC patients at Sichuan Cancer Hospital between February 1, 2024 and February 1, 2025. The study workflow is presented in **Supplementary Figure 1**, and the TRIPOD checklist is provided in **Supplementary Table S1**.

#### 2.3.1 Inclusion and exclusion criteria

Patients were eligible for enrollment if they met all of the following requirements: (1) a confirmed diagnosis of *de novo* mHSPC without any prior systemic anticancer therapy, in accordance with the EAU Guidelines on Prostate Cancer (16); (2) at least one lesion measurable under RECIST Version 1.1 criteria; (3) receipt of androgen deprivation therapy (ADT) plus an androgen receptor pathway inhibitor (ARPi), administered with or without docetaxel, as the initial systemic regimen; (4) an age between 18 and 85 years at enrollment together with signed informed consent on a voluntary basis; (5) and an Eastern Cooperative Oncology Group (ECOG) performance status no more than 2.

Patients were ineligible if any of the following applied: (1) a history of or concurrent additional primary malignancy of solid or hematologic origin; (2) missing or incomplete baseline imaging data (including chest computed tomography, abdominal ultrasonography, bone scintigraphy, brain magnetic resonance imaging, or PET-CT) or demographic and clinical records; (3) clinically significant concomitant conditions, such as cardiovascular or cerebrovascular disorders, severely compromised hepatic or renal function, or autoimmune disease; (4) ongoing active infection with hepatitis B or C virus; (5) pregnancy or breastfeeding; (6) or any documented immunodeficiency state.

Of the 230 patients initially screened, 157 met the eligibility criteria and were enrolled. Their data were subsequently randomized at a 7:3 ratio into a training cohort of 110 patients and a validation cohort of 47 patients; 103 and 44 patients in the two cohorts, respectively, completed the scheduled follow-up and constituted the final analytic population (**Figure 1**).

**Figure 1:**
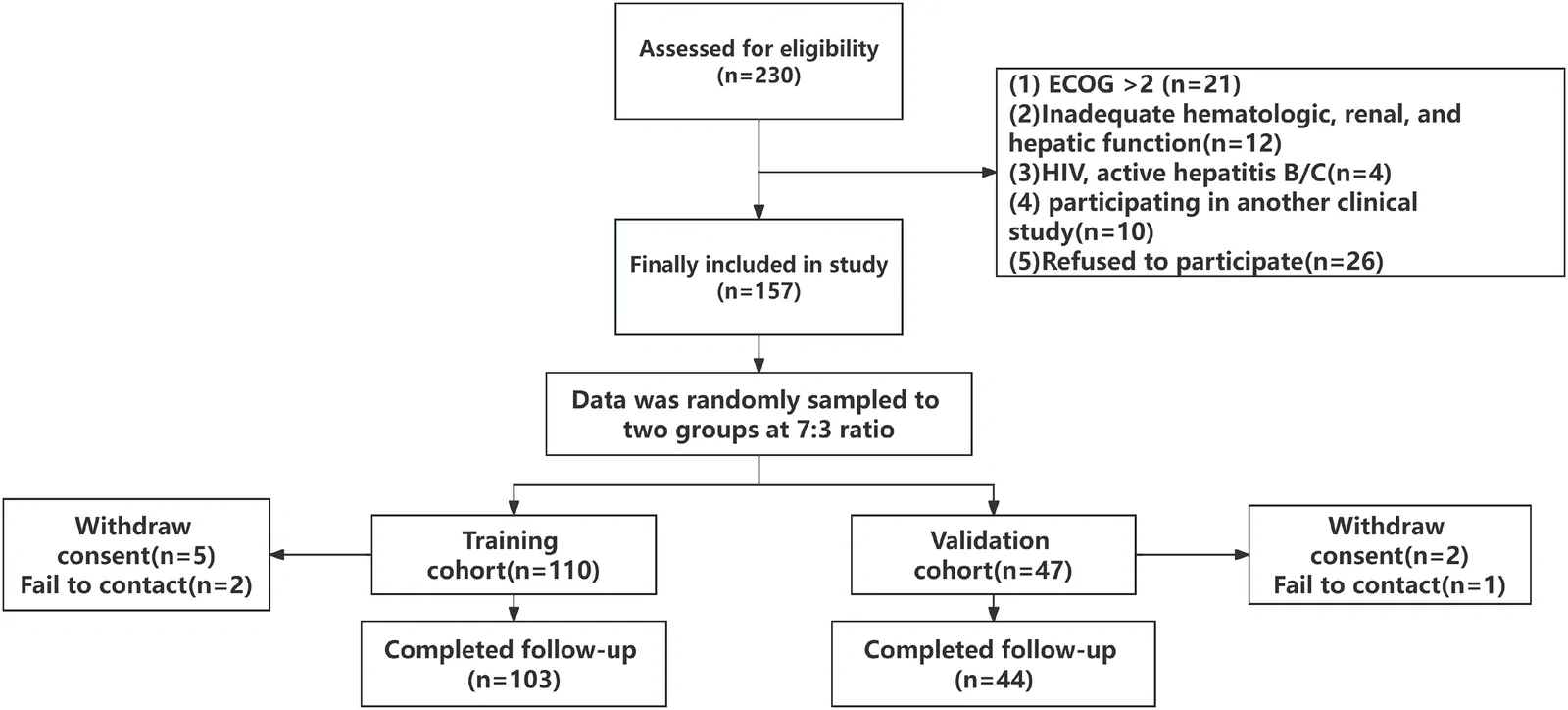
Patient screening flowchart.

#### 2.3.2 Sample size calculation

The sample size was estimated based on data from an East Asian cohort study of 496 patients with mHSPC receiving first-line therapy, in which 31% of patients progressed to mCRPC during a median follow-up of 18 months, with a median time to castration resistance of 10 months (17). Accordingly, we anticipated a cumulative event rate of 31% for progression to mCRPC during follow-up. Assuming a hazard ratio of 2.35 for the model, with a two-sided α of 0.05 and a statistical power of 80%, the Schoenfeld method yielded a minimum requirement of 44 events, corresponding to 142 participants. Allowing for approximately 10% attrition during follow-up, we therefore planned to enroll a minimum of 158 participants.

#### 2.3.3 Data collection and definitions

We collected the following data: age, pathological findings (including lymph node metastasis status), metastatic lesions, body weight, height, Gleason score, serial PSA measurements, imaging findings, and complete anti-tumor treatment information. The diagnosis of mCRPC was established according to the Prostate Cancer Clinical Trials Working Group 3 (PCWG3) criteria (6). Transcriptomic profiling was performed on coded samples by laboratory personnel who were blinded to clinical outcomes. Follow-up assessments, including PSA measurements, radiographic evaluations, and testosterone levels, were conducted by clinicians who were blinded to individual transcriptomic results. Progression to mCRPC was adjudicated by an independent team according to PCWG3 criteria, with adjudicators blinded to all molecular predictor data. All patients underwent imaging evaluations at the time of enrollment, at 12-week intervals throughout the initial year, and subsequently every 12 to 16 weeks, with additional scans performed whenever clinically warranted. The index date corresponded to the initiation of first-line therapy. mCRPC-free survival was the primary endpoint, calculated as the interval between the index date and the first documented diagnosis of mCRPC. When a patient switched to a subsequent line of therapy, the decision between progression and censoring relied on the most recent imaging study or PSA measurement obtained before that switch, and the progression date was assigned to the earliest time point at which any progression criterion was fulfilled. No missing data were observed for any baseline variables (age, BMI, PSA, Gleason score, ECOG performance status, alkaline phosphatase, hemoglobin, and metastatic burden). All enrolled patients had complete baseline assessments. Follow-up was conducted until June 1, 2026, yielding a median observation period of 611 days (95% CI: 560–641). Clinical data were collected during routine oncological care, encompassing scheduled outpatient consultations and telephone interviews, and were retrieved from the electronic medical record system. Participants remaining free of mCRPC were censored on the date of their most recent imaging examination or PSA determination. A patient was regarded as lost to follow-up when no clinic attendance, radiological record, or telephone contact was documented within 3 months prior to June 1, 2026, or upon formal withdrawal of consent for the use of personal data.

#### 2.3.4 Transcriptome sequencing

Total RNA was isolated from peripheral blood samples using TRIzol^®^ Reagent, with quality assessed by NanoDrop ND-2000 (for concentration and purity) and Agilent 5300 Bioanalyzer (for RNA integrity). All RNA libraries were constructed and sequenced in a single batch to minimize technical variability. As samples were not distributed across multiple sequencing runs or library preparation batches, formal batch effect correction was not required. Samples meeting thresholds of ≥20 ng/μL, >1 μg total yield, and RQN >4.5 were retained. Strand-specific libraries were prepared from 1 μg RNA samples that met quality thresholds using the Illumina^®^ Stranded mRNA Prep, Ligation kit, involving poly(A) selection, fragmentation, reverse transcription, and PCR amplification (10–15 cycles). Reference-guided transcriptome assembly and transcript abundance profiling were executed with StringTie; subsequently, RSEM was applied to derive gene-level expression counts (18, 19).

#### 2.3.5 Identification of intersection genes

To identify DEGs between groups, we performed differential expression analysis using the LIMMA R package (20). We set the thresholds for DEGs at P < 0.05 and absolute fold change > 1.5 and visualized the DEGs using volcano plots generated with ggplot2 in R. To identify genes associated with mCRPC, we intersected the mCRPC-related DEGs from our cohort and the specifically upregulated genes in mCRPC-related cell clusters from the GSE288878 dataset, ultimately obtaining genes strongly linked to mCRPC.

#### 2.3.6 Construction of a prognostic nomogram for mCRPC

The glmnet package (v4.0-2) was employed to perform elastic net regularization (α = 0.5), with the optimal penalty determined by 10-fold cross-validation using the λ.1se criterion. The λ.1se criterion yielded a parsimonious set of 3 predictors with nonzero coefficients, which were then refitted in a conventional multivariate Cox regression to derive the final prognostic model. Regression outputs, namely hazard ratios accompanied by 95% confidence intervals, were summarized in forest plots generated via ggplot2 (v3.5.1). The optimal risk-score cutoff for stratifying patients into high- and low-risk groups was determined using the maxstat package (v0.7-26) in R, which maximized the log-rank statistic for survival comparison between groups. This cutoff was derived exclusively from the development cohort and subsequently applied to the validation cohort without re-estimation. To facilitate clinical application, the fitted coefficients were used to construct a nomogram with the rms package (v4.4.0), enabling estimation of each patient’s probability of developing mCRPC at 365 days (1 year) and 545 days (approximately 1.5 years). Kaplan–Meier estimation and related survival procedures were performed using the survival (v3.6-4) and survminer (v0.4.9) packages. Model performance was examined from three complementary perspectives: discrimination, assessed by the time-dependent area under the curve (tdAUC); calibration, evaluated with calibration curves from the rms (v6.3-0) and riskRegression (v2025.05.20) packages; and clinical utility, investigated through decision curve analysis using the DCA (v1.0), ggDCA (v1.1), and dcurves (v0.5.0) packages, whereby the net benefit of model-guided decisions was compared across threshold probabilities, with superior strategies occupying higher positions on the plot. A binary cutoff was determined exclusively in the training cohort by matching the proportion classified as high risk to the proportion of LATITUDE-defined high-risk patients. Predictive performance at the prespecified 365-day horizon was compared using inverse probability of censoring weighting (IPCW), paired bootstrap resampling, and exact McNemar tests. Patients censored before 365 days were excluded from the complete-case McNemar analysis. Benjamini-Hochberg correction was applied for multiple comparisons.

### 2.4 Assessment of the prognostic value of marker genes

RNA sequencing data for prostate adenocarcinoma (PRAD) were downloaded from The Cancer Genome Atlas (TCGA) Data Portal (https://portal.gdc.cancer.gov/; last accessed on February 1, 2022). The final analytical cohort consisted of 498 tumor specimens. Raw FPKM values were first log2-transformed following the addition of a pseudo-count of 1. PFS served as the outcome. The TCGA database provided no explicit documentation regarding which samples represented mCRPC, so we used PFS as the outcome for this analysis. Stepwise Cox proportional hazards modeling was conducted to identify genes independently associated with PFS. The coxph function from the survival package (v3.5-5) was employed for model fitting, and the stepAIC function from the MASS package (v7.3-60) was utilized for variable selection.

### 2.5 Analysis of the potential mechanisms of prognostic genes in prostate cancer

#### 2.5.1 Cell viability assay

C4-2B cells (catalog no. SNL-161, Wuhan Sunncell Biotechnology, Wuhan, China) (5,000/well) were seeded with 100 μL complete medium and incubated for 24 hours. Cells were then treated with serial concentrations of Ompenaclid (0, 150, 300, or 450 μM). CCK-8 solution (10 μL/well; Bioss, China) was added at 6, 24, and 48 hours after treatment, and plates were incubated for 2 hours at 37 °C prior to measuring absorbance at 450 nm on a microplate reader.

#### 2.5.2 Annexin V-FITC/PI staining

Post-treatment, C4-2B cells were washed, resuspended in 1× binding buffer (1 × 10^6^ cells/mL), and stained with 5 μL Annexin V-FITC plus 5 μL PI per 100 μL suspension for 15 min at room temperature in the dark. Samples were diluted with 400 μL binding buffer and analyzed immediately by flow cytometry (Quanta SC, Beckman Coulter; CytExpert 2.3).

#### 2.5.3 Functional enrichment analysis on transcriptomic data

We used the R package clusterProfiler for GO and KEGG pathway analysis on DEGs between control cells and C4-2B cells treated with the selected concentration of Ompenaclid (21). GO analysis subsequently assigned DEGs into three principal classes—molecular function (MF), biological process (BP), and cellular component (CC)—followed by KEGG enrichment analysis to identify the signaling pathways in which these genes may participate.

#### 2.5.4 Weighted gene co-expression network analysis

We conducted WGCNA with the R package WGCNA (v1.72). Before constructing the network, we removed low-expression and low-variability genes by enforcing a minimum mean expression of 1 and a coefficient of variation ≥ 0.1. A signed co-expression network was then built via soft-thresholding; we selected power β = 10 because it achieved a scale-free topology fitting index (R²) approaching 0.9 while maintaining reasonable sparsity without excessive loss of connectivity. The adjacency matrix was transformed into a topological overlap matrix (TOM). Gene modules were delineated via the dynamic tree cut algorithm, with a minimum module size threshold of 30 genes. Modules with eigengene correlations ≥ 0.25 were consolidated (mergeCutHeight = 0.25), and only those with a minimum module eigengene connectivity (KME) of 0.3 were retained. To evaluate module-trait relationships, we computed Spearman correlation coefficients between module eigengenes and treatment groups. For network visualization, we selected the top 30 hub genes based on intramodular connectivity and displayed edges with a topological overlap weight > 0.02.

#### 2.5.5 Western blot analysis

Equal protein loads were resolved by SDS-PAGE and electroblotted onto PVDF membranes (Millipore). Following blocking with 5% skim milk in TBST, the membranes were probed with the indicated primary antibodies: HK2 (1:5000), SLC6A8 (1:1000), PFKP (1:5000), ALDH1B1 (1:4000), and β-Actin (1:4000). After washing, we incubated the membranes with HRP-linked secondary antibodies and developed the signals with high-sensitivity ECL (Thermo Fisher Scientific). Band intensities were quantified using ImageJ.

#### 2.5.6 Untargeted metabolomics approach

Cell pellets stored at −80 °C were thawed on ice and resuspended in 500 μL of prechilled methanol/water (4:1, v/v) supplemented with internal standards. After brief vortexing, metabolite extraction was enhanced by three cycles of freezing in liquid nitrogen for 5 min, incubation on dry ice for 5 min, and thawing on ice followed by 2 min of vortex mixing. Subsequently, a 200-μL aliquot was subjected to LC–MS/MS analysis. Chromatographic separation was achieved using a Waters ACQUITY Premier HSS T3 column (1.8 μm, 2.1 × 100 mm) maintained at 40 °C, with a 9-min gradient and a flow rate of 0.4 mL/min. The mass spectrometer was operated with spray voltages of 3.5 kV in positive mode and 3.2 kV in negative mode. The sheath and auxiliary gas settings were 30 and 5 arbitrary units, respectively, while the transfer tube and vaporizer temperatures were set at 320 and 300 °C. Stepped normalized collision energies of 30, 40, and 50 V were applied. Precursors exceeding an intensity threshold of 1 × 10^6^ were selected using a top-10 acquisition strategy with a 3-s exclusion period. Metabolites showing between-group differences were identified based on VIP > 1 and a Student’s t-test P < 0.05. OPLS-DA was performed in MetaboAnalystR using log_2_-transformed and mean-centered data, and model stability was evaluated by 200 permutation tests.

### 2.6 Statistical analysis

All analyses were conducted in R (version 4.4.1). Data distribution was evaluated using the Shapiro-Wilk test. Continuous variables with an approximately normal distribution are summarized as mean ± standard deviation and were analyzed using an independent-samples t-test for two-group comparisons or one-way ANOVA followed by Tukey’s *post hoc* test for comparisons involving more than two groups. Variables that did not meet the normality assumption are reported as median and interquartile range and were assessed using the Mann-Whitney U test or the Kruskal-Wallis test followed by Dunn’s test, as applicable. Categorical data are expressed as frequencies and percentages and were evaluated using the chi-square test or Fisher’s exact test. Longitudinal CCK-8 measurements were analyzed using a linear mixed-effects model accounting for repeated observations. All tests were two-sided, with statistical significance defined as P < 0.05. Bonferroni adjustment was used when correction for multiple testing was required (22–25).

## 3 Results

### 3.1 Single-cell transcriptomic analysis identified two tumor cell clusters in the GSE288878 CRPC model

Five distinct cell clusters (clusters 0–4) were identified and visualized via t-SNE and UMAP dimensionality reduction (**Figures 2a, b**). The top five marker genes characterizing each cluster are presented as dot plots (**Figure 2c**). As shown in the stacked bar chart (**Figure 2d**), clusters 1, 2, and 4 predominated in the GSM8777871 sample derived from hormone-sensitive prostate cancer (HSPC), whereas clusters 0 and 3 constituted the major populations in the GSM8777872 sample from mCRPC. We employed model-based differential expression analysis (Seurat FindAllMarkers) by comparing each cell cluster against all other clusters to identify cluster-specific marker genes. Volcano plots are shown in **Figure 2e**. Subsequent scMetabolism profiling demonstrated that clusters 0 and 3 served as the primary contributors to propanoate metabolism, galactose metabolism, glycolysis/gluconeogenesis, and pyruvate metabolism (**Figure 2f**).

**Figure 2:**
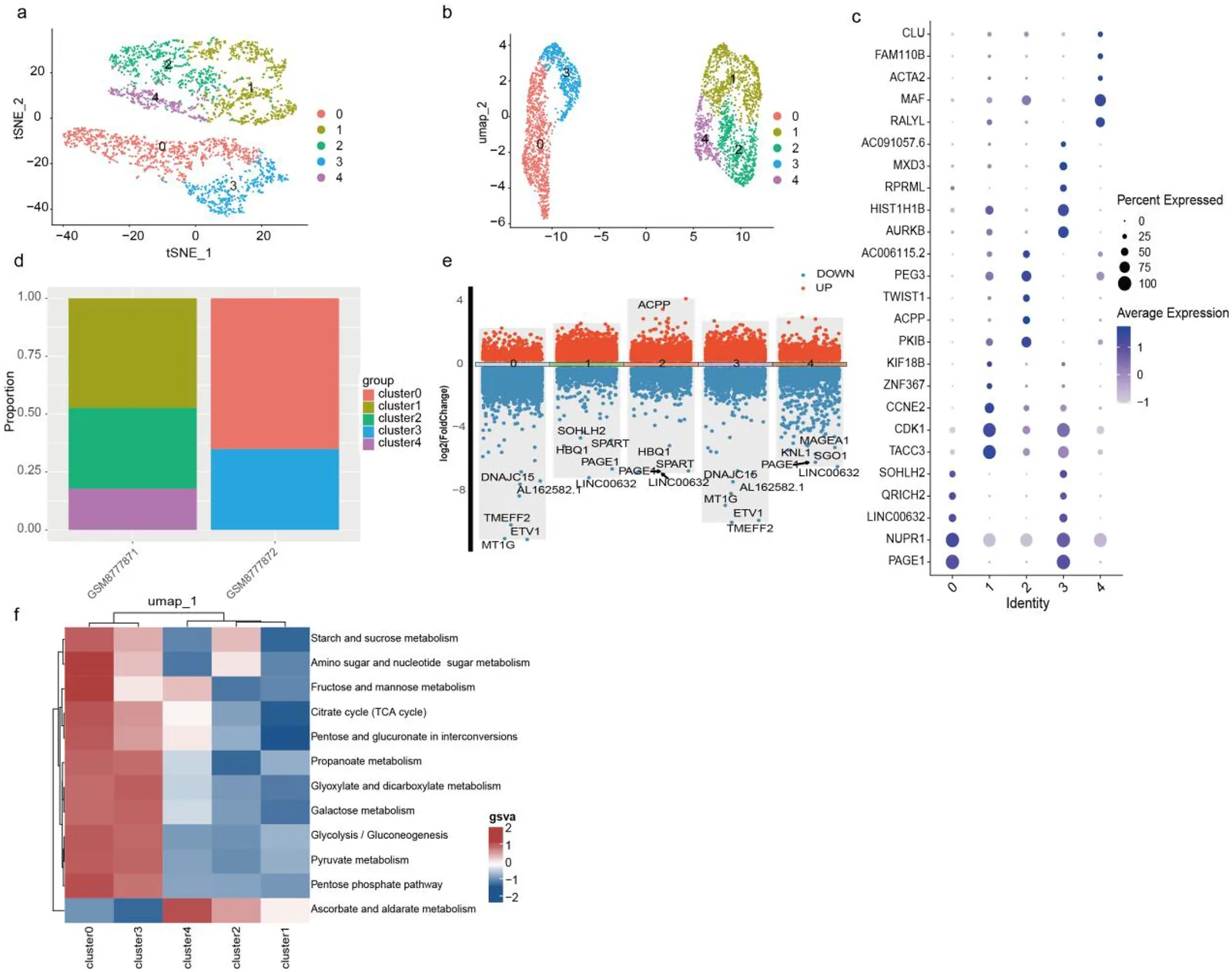
Single-cell transcriptomic profiling reveals distinct cellular clusters and metabolic heterogeneity in the LNCaP-FGC and LNCaP-C4-2B cell models.t-SNE visualization of five identified cell clusters (clusters 0–4). **(b)** UMAP visualization of the same cell clusters, confirming the robustness of the clustering pattern. **(c)** Dot plot showing the top five marker genes for each cluster. **(d)** Stacked bar chart illustrating the proportional distribution of cell clusters in GSE8777871 and GSE8777872. **(e)** Volcano plots were used to visualize specifically upregulated genes in each cluster. **(f)** Heatmap of scMetabolism analysis displaying the activity scores of representative metabolic pathways across the five clusters.

### 3.2 Five DEGs closely related to mCRPC were identified

A total of 147 patients completed follow-up in this prospective cohort study, with 54 cases progressing to mCRPC during follow-up. Baseline characteristics of patients in the two cohorts are compared in **Table 1**.

**Table 1:** Comparison of baseline characteristics between the development and validation cohorts.

Variable	Development set (n=103)	Validation set (n=44)	Statistics value	P value
BMI	23.47 ± 4.44	23.80 ± 3.72	-0.47	0.64
Age	70.41 ± 8.26	69.93 ± 9.36	0.29	0.77
Gleason score			0.12	0.73
≤8	61 (59.22%)	24 (54.55%)		
>8	42 (40.78%)	20 (45.45%)		
Treatment			0.02	0.90
ADT+ARPI	52 (50.49%)	21 (47.73%)		
ADT+ARPI+Docetaxel	51 (49.51%)	23 (52.27%)		
Lymph node metastasis	49 (47.57%)	15 (34.09%)	1.76	0.18
ECOG PS			0.47	0.49
<2	43 (41.75%)	15 (34.09%)		
2	60 (58.25%)	29 (65.91%)		
Total PSA	47.014 ± 65.651	41.166 ± 49.736	0.591	0.556
Bone metastasis burden			1.554	0.213
<3 sites	69 (67.0%)	24 (54.5%)		
≥ 3 sites	20 (45.5%)	20 (45.5%)	1.554	0.213
Measurable visceral metastasis	36 (35.0%)	21 (47.7%)	0.616	0.204
LDH	210.442 ± 47.731	210.570 ± 40.781	-0.017	0.987
ALP	91.858 ± 28.230	91.918 ± 24.042	-0.013	0.99
Hb	123.755 ± 13.111	124.182 ± 12.332	-0.188	0.851

We conducted transcriptome sequencing on peripheral blood specimens from patients in our cohort. Compared with the control group, 233 upregulated and 293 downregulated genes were identified in the mCRPC group (**Figures 3a, b**). By intersecting the DEGs from our cohort, and the characteristic genes of CRPC-related cell types from the GSE288878 dataset, we obtained a total of 5 genes, including SLC25A39, GRB10, SELENBP1, SLC6A8, and YBX3 (**Figure 3c**).

**Figure 3:**
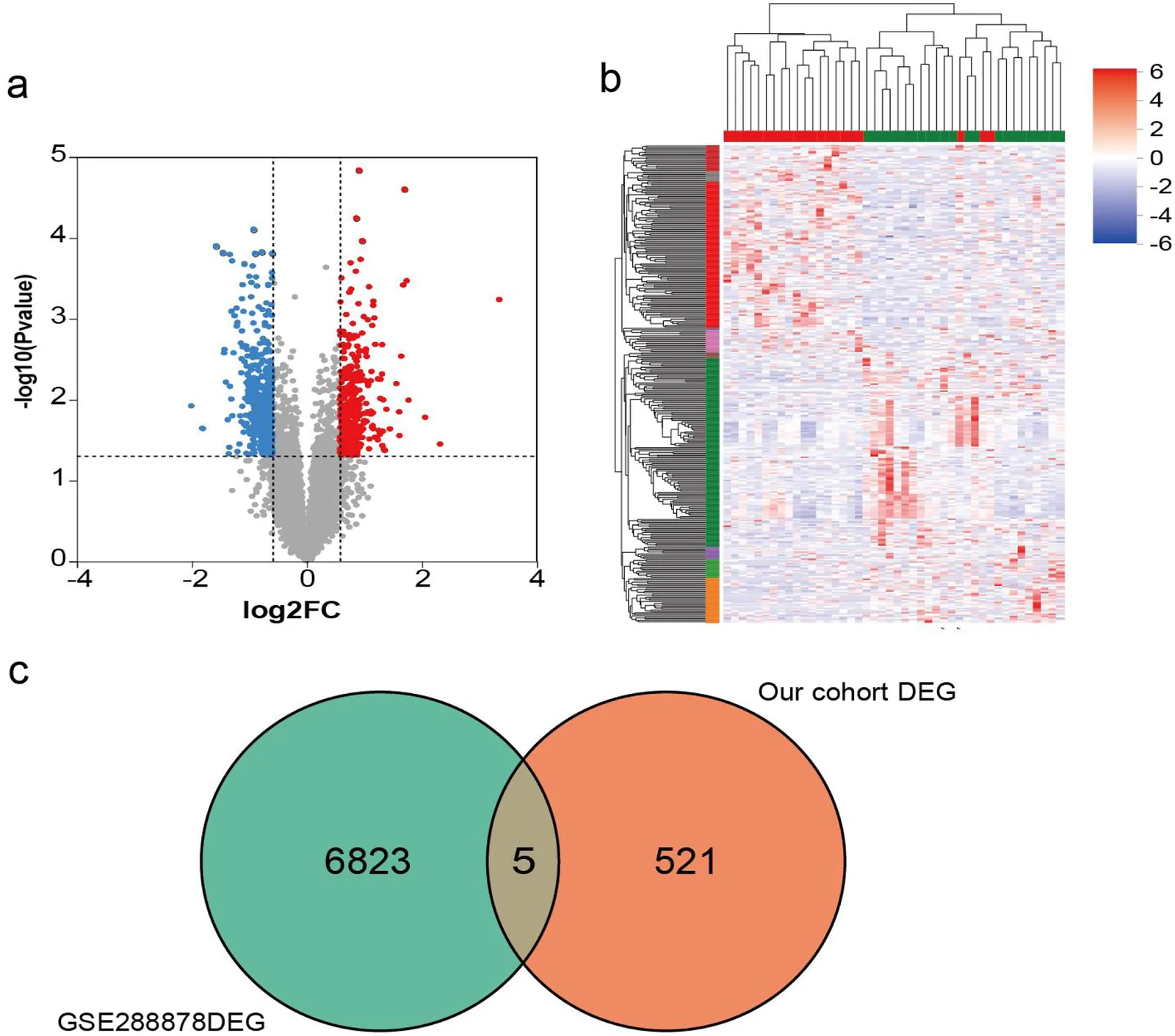
A shared gene signature linked to CRPC-related cell types was extracted from the overlap among DEGs identified in our cohort and GSE288878 datasets, yielding five overlapping genes: SLC25A39, GRB10, SELENBP1, SLC6A8, and YBX3. **(a)** Volcano plot of DEGs in our cohort; **(b)** heatmap for DEGs; **(c)** Venn diagram identifying the final five intersection genes).

### 3.3 A prognostic model was developed to forecast the transition from mHSPC to mCRPC

The optimal tuning parameter (λ) was determined via 10-fold cross-validation to further ensure model stability. Elastic Net regularization retained three variables with non-zero coefficients were retained at λ.1se, namely SLC25A39 (0.0438), SLC6A8 (0.131), and YBX3 (0.069) (**Figures 4a, b**). All 3 variables satisfied the proportional hazards assumption (P > 0.05 for each, Schoenfeld residual test). The variance inflation factor (VIF) values for SLC25A39, SLC6A8, and YBX3 were 1.30, 1.19, and 1.47, respectively, indicating no multicollinearity among these three variables. In the multivariate Cox model, SLC25A39 and SLC6A8 emerged as independent predictors of progression to mCRPC (**Figure 4c**). The resulting model was presented graphically as a nomogram (**Figure 4d**), yielding C-index values of 0.849 (95% CI: 0.795–0.899) in the development cohort and 0.783 (95% CI: 0.688–0.880) in the validation cohort; time-dependent AUCs are depicted in **Figures 4e, f**. The prognostic score was computed as: risk score = 0.460 × SLC25A39 + 0.539 × SLC6A8 + 0.374 × YBX3. Using the maxstat package (version 0.7-25) in R, the optimal cutoff value for the risk score was 11.356. Patients were subsequently stratified into high- and low-risk groups. High-risk scores were significantly associated with accelerated progression to mCRPC (HR = 17.19, 95% CI: 6.55-45.11, P < 0.0001; **Figure 4g**). The median mCRPC-free survival was 466 days in the high-risk group, whereas it was not reached in the low-risk group, with more than half of the patients remaining free from mCRPC progression at the end of follow-up. The model demonstrated an AUC of 0.89 (95% CI: 0.80–0.97) for predicting 1-year mCRPC-free survival in the development cohort (**Figure 4e**), and 0.83 (95% CI: 0.62–1.00) in the validation cohort (**Figure 4f**). The predicted and observed mCRPC probabilities exhibited strong concordance, as illustrated by calibration curves for both the development and validation (**Figure 4h**) datasets, indicating robust agreement between estimated and actual outcomes. In the development cohort, the calibration intercept was −0.018, and the calibration slope was 1.553, suggesting modest under-confidence of the predicted probabilities. The Brier score was 0.056, reflecting favorable overall predictive accuracy. In the internal validation cohort, the calibration intercept was 0.093 and the calibration slope was 1.274, both approaching the ideal values of 0 and 1, respectively. The Brier score in the validation cohort was 0.060, comparable to that of the development cohort. Decision curve analysis subsequently evaluated the net clinical benefit across a range of risk thresholds, substantiating the nomogram’s practical value in informing treatment strategies for patients in both cohorts (**Figure 4i**). The model demonstrated superior net benefit compared with both the “treat-all” and “treat-none” strategies at threshold probabilities ranging from approximately 0.2 to 0.6, indicating favorable clinical utility across a broad spectrum of clinically relevant decision thresholds. At both evaluation time points, the three-gene model demonstrated significantly higher specificity and IPCW-adjusted accuracy than LATITUDE. At 365 days, the three-gene model achieved higher specificity (82.1% vs. 51.3%; adjusted P = 0.016) and accuracy (81.0% vs. 52.4%; adjusted P = 0.018). At 545 days, the three-gene model also showed higher specificity (94.7% vs. 26.3%; adjusted P < 0.001), accuracy (81.1% vs. 29.3%; adjusted P < 0.001), and balanced accuracy (75.0% vs. 30.6%; adjusted P = 0.001). In contrast, sensitivity did not differ significantly between the three-gene model and LATITUDE at either 365 days (67.2% vs. 66.4%; adjusted P = 0.988) or 545 days (55.3% vs. 34.9%; adjusted P = 0.474). The difference in balanced accuracy at 365 days was also not statistically significant (74.6% vs. 58.8%; adjusted P = 0.648).

**Figure 4:**
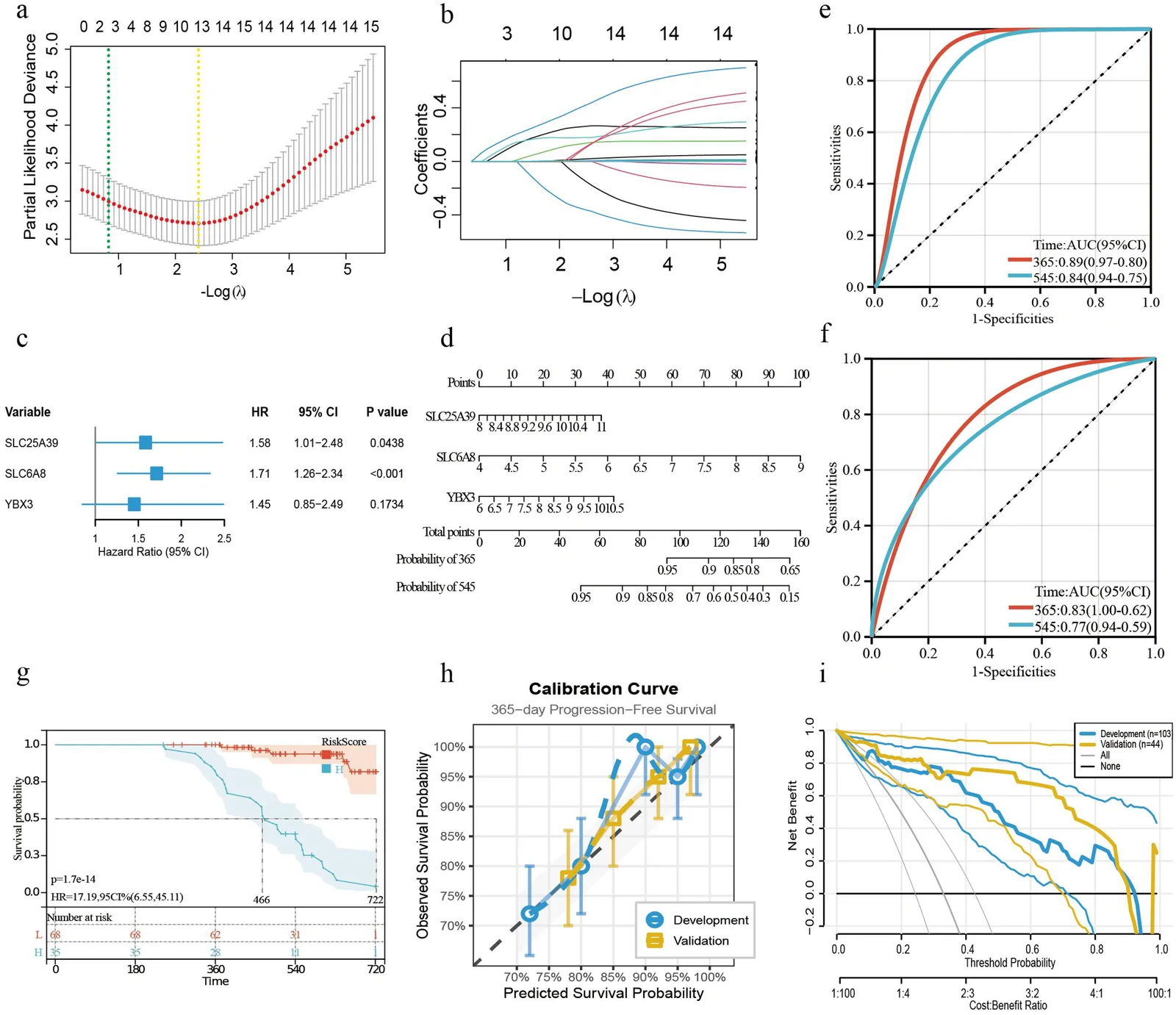
Development and internal validation of an Elastic Net-Cox prognostic signature for mCRPC. **(a)** Ten-fold cross-validation profile of partial-likelihood deviance plotted against log(λ); vertical dashed lines denote λ.1se (green) and λ.min (yellow). **(b)** Regularization paths illustrating the shrinkage of individual coefficients as the penalty parameter increases. **(c)** Forest plot of hazard ratios and 95% confidence intervals from the multivariable Cox model incorporating Elastic Net-selected variables. **(d)** A three-predictor nomogram for estimating mCRPC-free survival probabilities at 365 and 545 days; individual predictor scores are summed to yield the total risk score and corresponding survival estimate. **(e, f)** Time-dependent receiver operating characteristic (ROC) curves assessing discriminative performance in the training cohort **(e)** and the validation cohort, respectively. **(g)** Kaplan-Meier survival estimates. **(h)** Calibration plots comparing predicted and observed mCRPC-free survival probabilities across both cohorts. **(i)** DCA evaluating the net clinical benefit.

### 3.4 Prognostic value assessment of five genes in prostate cancer tissues

We performed stepwise Cox regression analysis on the five intersection genes using tumor specimens from all prostate cancer patients in the TCGA database. Since the TCGA database does not specify which samples are CRPC cases, progression-free survival (PFS) was adopted as the primary endpoint. Stepwise Cox regression identified SLC6A8 as an independent risk factor for PFS (**Figure 5**).

**Figure 5:**
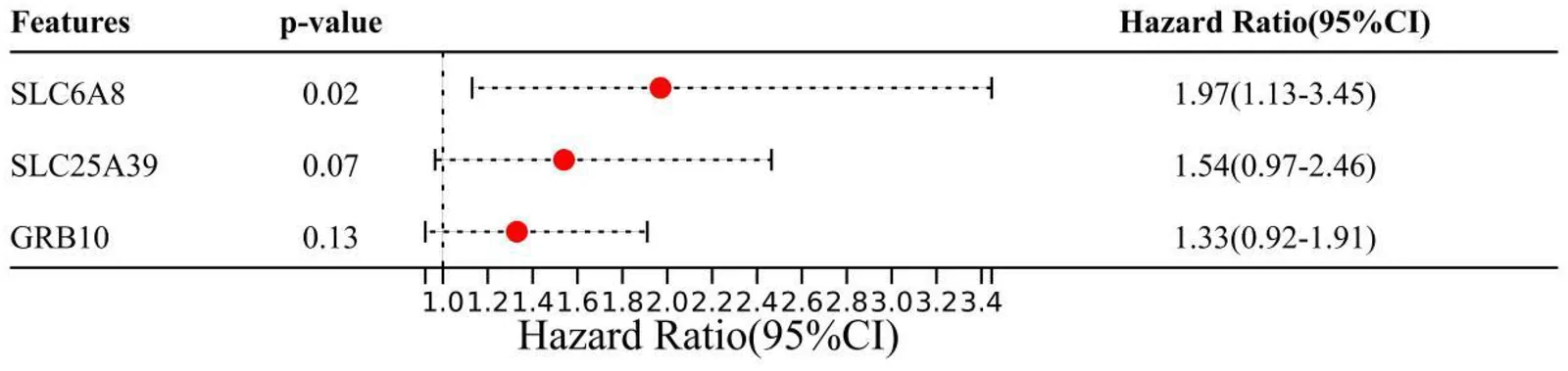
Forest plot for stepwise Cox regression.

### 3.5 SLC6A8 may promote CRPC cell proliferation by enhancing glycolysis

#### 3.5.1 Ompenaclid, an SLC6A8 inhibitor, inhibited proliferation and promoted apoptosis in prostate cancer cells

C4-2B cells were incubated with the SLC6A8 inhibitor ompenaclid to probe its role in prostate cancer. CCK-8 assays indicated dose-dependent viability suppression at 48 h (**Figure 6a**), while Annexin V/PI flow cytometry showed corresponding increases in early and late apoptotic cells across 0–450 μM (**Figures 6b, c**).

**Figure 6:**
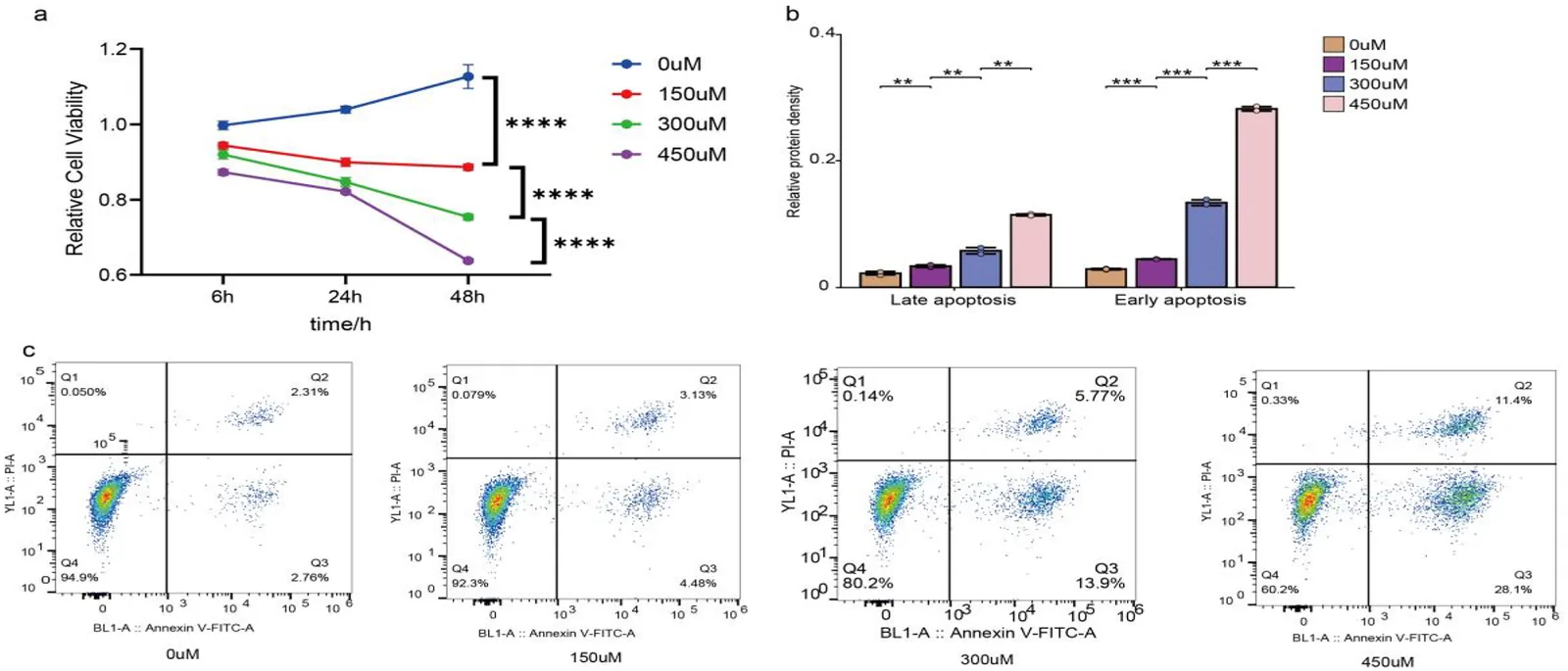
Ompenaclid inhibited proliferation and promoted apoptosis in C4-2B cells. **(a)** Dose-dependent suppression of C4-2B proliferation by Ompenaclid was confirmed by CCK-8 assay at 48 h. **(b)** Box plots comparing the effects of different Ompenaclid concentrations on early and late apoptosis in C4-2B cells. **(c)** Representative cytograms of flow cytometry for each Ompenaclid dose.

#### 3.5.2 Ompenaclid at 450 μM inhibited glycolysis in prostate cancer cells

We selected 450 μM Ompenaclid to treat C4-2B cells for 48 hours and performed transcriptome sequencing on both the treated and control groups. In C4-2B cells exposed to Ompenaclid, transcriptomic profiling revealed 2,874 upregulated and 1,899 downregulated transcripts relative to the control group (**Figure 7a**). For weighted gene co-expression network construction, the scale-free topology criterion was evaluated across a spectrum of soft-thresholding powers; a β value of 10 yielded an optimal scale-free topology fitting index (R²) and was therefore adopted for subsequent analyses (**Figure 7b**). This power yielded a reasonably sparse network without excessive loss of connectivity (**Figure 7c**). Nine distinct gene modules emerged from the hierarchical cluster analysis of the weighted co-expression network, with their inter-module relationships visualized through the dendrogram shown in **Figure 7d**. Module-trait associations were assessed by Pearson correlation of eigengenes with treatment status (**Figure 7e**). The turquoise module correlated positively with Ompenaclid (r = 0.87, P = 0.00106), whereas the blue module correlated negatively with drug treatment (r = −0.87, P = 0.00106), indicating control group enrichment. The Venn diagram in **Figure 7f** showed the intersection of upregulated DEGs in the Ompenaclid group and WGCNA turquoise module genes, yielding 1,072 overlapping upregulated genes. The Venn diagram in **Figure 7g** showed the intersection of downregulated DEGs in the Ompenaclid group and WGCNA blue module genes, yielding 638 overlapping downregulated genes. Biological process enrichment analysis of the 1,072 overlapping upregulated genes revealed enrichment in system development, biological regulation, anatomical structure morphogenesis, nervous system development, and cell-cell signaling (**Figure 7h**). The cellular component GO terms significantly enriched in the target gene set included cell projection, synapse, and neuron projection (**Figure 7i**). Correspondingly, the molecular function ontology highlighted enrichment in cation binding, metal ion binding, enzyme regulator activity, and ion channel activity (**Figure 7j**). KEGG pathway enrichment analysis identified significant enrichment in herpes simplex virus 1 infection, Ras signaling pathway, glycerophospholipid metabolism, phospholipase D signaling pathway, and PPAR signaling pathway (**Figure 7k**). For downregulated genes, biological process ontology revealed significant overrepresentation in cellular macromolecule biosynthetic process, cellular nitrogen compound biosynthetic process, and response to stress (**Figure 7l**). Within the cellular component category, enriched terms encompassed protein-containing complex, nuclear lumen, endomembrane system, nucleoplasm, organelle membrane, and endoplasmic reticulum (**Figure 7m**). Molecular function analysis highlighted catalytic activity, cation binding, carbohydrate derivative binding, and DNA binding (**Figure 7n**). Additionally, KEGG pathway mapping identified significant enrichment in TGF-beta signaling pathway, cell cycle, mineral absorption, DNA replication, homologous recombination, and glycolysis/gluconeogenesis (**Figure 7o**). Notably, HK2, PFKP, and ALDH1B1 were genes enriched in the glycolysis/gluconeogenesis signaling pathway. Western blot analysis confirmed that Ompenaclid downregulated the expression of SLC6A8, HK2, PFKP, and ALDH1B1 in C4-2B cells in a dose-dependent manner, with 450 μM Ompenaclid exerting the strongest inhibitory effect on these four genes compared with other concentrations, suggesting optimal suppression of glycolysis. Accordingly, we performed untargeted metabolomic sequencing in the control and 450 μM Ompenaclid groups. Unsupervised PCA revealed distinct clustering patterns between the two groups (**Figure 7r**). Supervised PLS-DA and OPLS-DA further accentuated this metabolic separation, indicating pronounced metabolomic alterations following Ompenaclid intervention (**Figures 7s, t**). Compared with control C4-2B cells, 450 μM Ompenaclid treatment resulted in elevated levels of 629 metabolites and decreased levels of 279 metabolites (**Figure 7u**). The significant downregulation of reduced glutathione, lactic acid, 4-amino-7-chloroquinoline, 3-chloro-L-tyrosine, L-gulose, pyruvic acid, fructose-1,6-bisphosphate, as well as glucose-6-phosphate, dulcitol, and glucuronic acid in the 450 μM Ompenaclid group suggested that 450 μM Ompenaclid suppressed glycolysis in CRPC cells(**Figure 7v**). We selected the top 50 metabolic sets ranked by P-value for MSEA enrichment analysis and visualization. The results revealed that differential metabolites were significantly enriched in pathways including glycolysis/gluconeogenesis and phenylalanine, tyrosine and tryptophan biosynthesis (**Figure 7w**).

**Figure 7:**
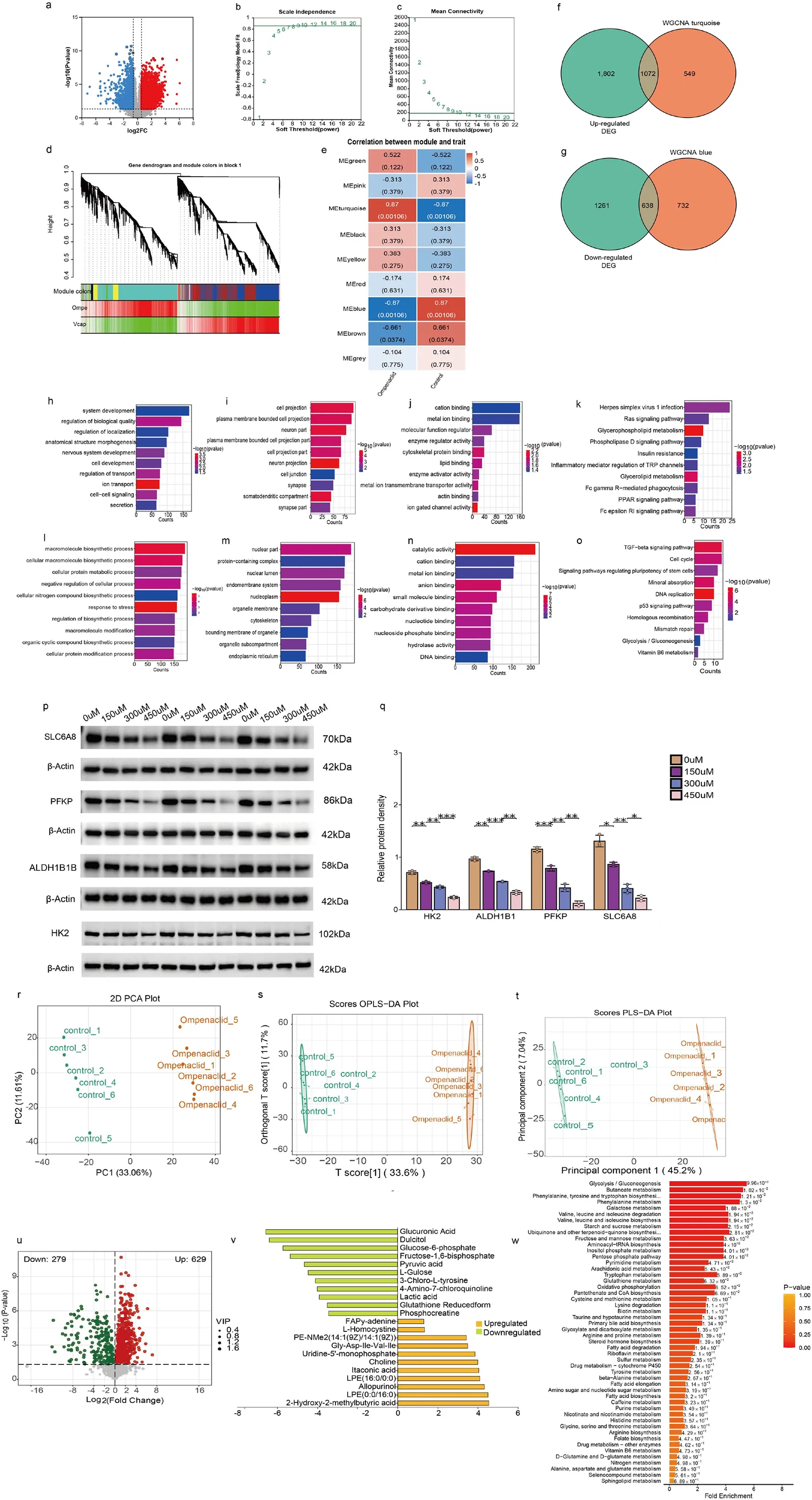
Ompenaclid suppressed glycolysis in C4-2B cells, which was associated with downregulation of SLC6A8, HK2, PFKP, and ALDH1B1. **(a)** Volcano plot depicted DEGs. **(b)** The soft-thresholding power (β) was optimized for scale-free network topology. **(c)** Connectivity distribution at β = 10 demonstrated adequate network sparsity while preserving sufficient node connectivity. **(d)** Unsupervised hierarchical clustering of the co-expression network partitioned the transcriptome into nine discrete modules. **(e)** Heatmap of Pearson correlations between module eigengenes and experimental conditions was generated. The turquoise module eigengene was robustly associated with Ompenaclid exposure (r = 0.87, P = 0.00106), whereas the blue module eigengene displayed an inverse relationship with drug treatment (r = −0.87, P = 0.00106) and was thus tightly coupled to the control phenotype. **(f)** Venn diagram illustrated the overlap between upregulated DEGs in the Ompenaclid-treated group and genes assigned to the WGCNA turquoise module, identifying 1,072 convergent upregulated genes. **(g)** Venn diagram showing the intersection of DEGs in the Ompenaclid group and WGCNA blue module genes, yielding 638 overlapping downregulated genes. **(h–k)** GO and KEGG enrichment analysis of 1,072 overlapping upregulated genes. **(h)** BP enrichment. **(i)** CC enrichment. **(j)** MF enrichment. **(k)** KEGG pathway enrichment. **(l–o)** GO and KEGG enrichment analysis of 638 overlapping downregulated genes. **(l)** Biological process enrichment. **(m)** Cellular component enrichment. **(n)** Molecular function enrichment. **(o)** KEGG pathway enrichment, with HK2, PFKP, and ALDH1B1 enriched in the glycolysis/gluconeogenesis pathway. **(p)** Western blot analysis showing dose-dependent downregulation of SLC6A8, HK2, PFKP, and ALDH1B1 protein expression by Ompenaclid in C4-2B cells, with 450 μM exerting the optimal inhibitory effect. **(q)** Quantification of Western blot results. **(r)** PCA score plot **(s)** PLS-DA score plot. **(t)** OPLS-DA score plot. **(u)** Volcano plot of differential metabolites. **(v)** Bidirectional bar chart showing top 11 upregulated and top 11 downregulated metabolites in Ompenaclid-treated C4-2B cells. **(w)** Results of MSEA enrichment analysis.

## 4 Discussion

Several miRNAs have been identified as potential biomarkers for distinguishing CRPC from non-CRPC. Santos et al. analyzed 43 Portuguese patients with prostate cancer and found that those with high-Gleason-score tumors concomitantly exhibiting elevated miR-7 expression progressed to CRPC more rapidly (10 vs. 46 months) (26). Puente-Rivera et al. found that serum miR-107 levels were markedly elevated in CRPC patients compared to NCRPC patients; however, the diagnostic or prognostic utility of this miRNA for discriminating between mCRPC and mHSPC remains to be elucidated (27). Natalia Jiménez et al. examined the prognostic value of tumor suppressor genes (TSG) RB1, PTEN, and TP53 expression in tumor specimens from mHSPC patients, revealing that a TSG-low signature (19.2%) served as an independent predictor of abbreviated CRPC-FS (HR = 1.8, P = 0.002) (28). Marín-Aguilera et al. reported that peripheral blood TMPRSS2-ERG positivity correlated with resistance to docetaxel resistance in patients with mCRPC (29).

Several prognostic models have been developed to identify patients with mHSPC who are at increased risk of rapid progression to mCRPC (30–32). Earlier work has shown that merging routine clinicopathological features with biochemical markers can support individualized risk classification. Yet the generalizability of such approaches is hampered by limited sample size and predictive performance, retrospective study designs, the necessity for invasive specimen collection, and treatment algorithms that inadequately reflect present-day first-line mHSPC care. To the best of our knowledge, prognostic models integrating peripheral blood gene expression profiles with clinical variables to predict progression to mCRPC in mHSPC patients receiving first-line ADT-based therapy remain unavailable. In this study, we sought to construct a prognostic model for mCRPC, based on five intersecting genes (SLC25A39, GRB10, SELENBP1, SLC6A8, and YBX3). It should be acknowledged that bulk RNA sequencing of whole peripheral blood does not permit unambiguous attribution of detected transcripts to discrete cellular origins. The altered expression of five genes observed in our cohort could plausibly emanate from circulating tumor cells, tumor-derived extracellular vesicles, leukocyte-mediated host responses to malignancy, or an admixture thereof—possibilities that remain to be disentangled in subsequent studies.

Subsequently, we incorporated the five candidate genes together with ECOG performance status, Gleason score, age, BMI, and other clinical parameters as potential predictive variables. Elastic Net regularization identified three predictors: SLC25A39, SLC6A8, and YBX3. The results revealed that patients in the high-risk group exhibited a median mCRPC-free survival of 466 days (approximately 15.5 months), whereas in the low-risk group, the median mCRPC-free survival was not reached by the end of follow-up. We further constructed a nomogram to assist clinicians in estimating 365-day and 545-day (1.5-year) mCRPC-free survival probabilities for individual patients. Our transcriptomic signature offers a framework for precision risk stratification that could inform treatment decisions. First, for patients in whom clinical risk is equivocal—such as those with oligometastatic bone disease or borderline PSA levels—the signature could guide intensification with triplet therapy or earlier combination with local treatment (e.g., metastasis-directed radiotherapy) if the molecular profile predicts rapid progression. Second, in patients with high-volume disease but a favorable molecular signature, clinicians might consider omitting docetaxel to avoid chemotherapy-related toxicity while maintaining vigilant PSA and imaging surveillance, effectively personalizing the risk–benefit calculus. Third, the signature could serve as an enrichment tool for clinical trials, identifying mHSPC patients with aggressive molecular phenotypes who are most likely to benefit from novel therapeutic strategies (e.g., PARP inhibitor combinations, PSMA-targeted agents, or immunotherapy) before the development of castration resistance. These applications remain hypothetical pending prospective validation. The transition from a research biomarker to a clinically actionable test requires demonstration that risk-adaptive management improves clinically meaningful endpoints, such as time to mCRPC.

GRB10 expression in tumor tissue has been implicated in the progression of CRPC and resistance to ARPIs. In clinical prostate cancer specimens, elevated GRB10 expression was associated with inferior patient outcomes. GRB10 knockdown significantly attenuated prostate cancer cell proliferation and AKT activity, a well-established mediator of CRPC (33). In our study, elevated peripheral blood expression of SLC25A39 and SLC6A8 were identified as independent risk factors associated with accelerated progression to mCRPC. SLC25A39 mediates GSH transport into mitochondria, thereby contributing to the inhibition of cuproptosis and ferroptosis in cancer cells (34, 35). In hepatocellular carcinoma, SLC25A39 promotes sorafenib resistance by attenuating mitochondrial oxidative stress and subsequently blocking ferroptosis (36). SLC25A39 protein abundance increases after GSH depletion without a corresponding change in transcript levels, suggesting post-transcriptional regulation of mitochondrial GSH transport (37). Heng et al. identified an association between SLC25A39 and prostate cancer; however, the relationship between SLC25A39 and CRPC remains to be further investigated (38). SELENBP1 exhibits stage-dependent effects in prostate cancer progression. As a methanethiol oxidase, it generates H_2_S and H_2_O_2_, which suppress mitochondrial respiration and activate AMPK, thereby reducing ATP production. In hormone-sensitive settings, this energy-limiting state may constrain malignant proliferation. However, in castration-resistant contexts, chronic AMPK activation might enhance metabolic flexibility, including compensatory upregulation of glycolysis and fatty acid oxidation, potentially supporting cell survival under androgen-deprived conditions (39–41). SLC6A8 facilitates cellular uptake of creatine and phosphocreatine to sustain the creatine-phosphocreatine energy shuttle in high-energy-demand tissues. Isotope tracing demonstrated that prostate cancer cells rely primarily on extracellular creatine uptake rather than *de novo* synthesis from arginine. SLC6A8 knockdown markedly lowered intracellular creatine and suppressed clonogenic growth of human prostate cancer cells (42).

Further interrogation of TCGA prostate cancer datasets demonstrated that elevated SLC6A8 expression was associated with shorter PFS in our study. Accordingly, we performed cell-based experiments to investigate the role and underlying mechanisms of SLC6A8 in prostate cancer cells by pharmacological inhibition. The anti-tumor efficacy of Ompenaclid has been benchmarked against genetic SLC6A8 pathway ablation in preclinical models, with comparable degrees of tumor growth inhibition and metastasis suppression (e.g., 83% vs. 86% liver metastasis inhibition in CRC models) (43).

Dose-dependent reductions in cell proliferation and increases in apoptotic cell death were observed following SLC6A8 inhibitor treatment. Notably, the 450 μM concentration exhibited superior efficacy compared with the other concentrations; therefore, we selected this concentration for subsequent transcriptomic and metabolomic profiling of SLC6A8 inhibitor-treated versus control cells. Through integrative differential expression analysis and WGCNA of transcriptomic data, we identified that genes downregulated in the SLC6A8 inhibitor group were significantly enriched in glycolysis-related signaling pathways, suggesting that the pro-tumorigenic role of SLC6A8 might be mechanistically linked to enhanced glycolysis. The specific genes included HK2, PFKP, and ALDH1B1. Tumor cells frequently rely on glycolysis rather than oxidative phosphorylation for energy generation, with glucose ultimately converted into lactate. This metabolic reprogramming constituted a fundamental biochemical hallmark of malignancy, widely recognized as the Warburg effect. HK2 catalyzed the initial committed step of glycolysis through phosphorylation of glucose to glucose-6-phosphate, thereby initiating glucose entry into the glycolytic cascade and facilitating the Warburg phenotype in cancer cells (44). PFKP, which encoded the platelet-specific isoform of phosphofructokinase, functioned as a critical rate-limiting enzyme that catalyzed the conversion at the third committed step of glycolysis, thereby governing overall glycolytic flux (45). Although ALDH1B1 is not a classical glycolytic enzyme, it indirectly supports glycolytic phenotypes by maintaining NAD^+^/NADH redox balance and detoxifying toxic aldehydes such as methylglyoxal, a byproduct of glycolysis. Collectively, these three genes may support glycolytic reprogramming, with HK2 and PFKP directly governing glycolytic flux and ALDH1B1 providing the redox homeostasis necessary for sustained glycolytic activity in tumor cells. Glycolytic intermediates and the terminal metabolite lactate were coordinately downregulated following 450 μM SLC6A8 inhibitor treatment, as revealed by subsequent metabolomic profiling. These findings collectively indicated that pharmacological SLC6A8 suppression impaired glycolytic flux in prostate cancer cells.

This study has several limitations. First, the single-center design at a tertiary hospital in China inevitably introduces selection bias. It should be emphasized that the entire cohort comprised East Asian patients; accordingly, whether the genetic architecture, environmental exposures, and tumor biological features inherent to this population can be generalized to Western or other ethnic groups remains untested. Specifically, the risk stratification thresholds established herein may not be directly transportable to non-Asian cohorts, and any cross-ethnic extrapolation should therefore be approached with circumspection. In addition, exhaustive searches of publicly accessible GEO repositories failed to identify any peripheral blood transcriptomic dataset from mHSPC patients with longitudinal follow-up suitable for external validation. The overwhelming majority of available expression data derive from cell lines or solid tumor tissues, which are fundamentally discordant with the blood-based molecular signature we developed. To redress this deficiency, a prospective multicenter validation initiative is currently being planned, with the aim of enrolling independent peripheral blood cohorts across three to five tertiary centers distributed in geographically distinct regions of China. The primary validation objectives encompass: (i) evaluating discriminative capacity via C-index and time-dependent AUC for mCRPC-free survival; (ii) assessing calibration fidelity at the 1-year and 1.5-year landmarks; (iii) quantifying net clinical benefit through decision curve analysis; and (iv) examining the robustness of the predefined risk threshold within subgroups defined by age, baseline PSA, and Gleason score. Attainment of these benchmarks is regarded as a prerequisite for any subsequent clinical translation. Second, the postulated mechanistic nexus between SLC6A8 and glycolytic reprogramming during CRPC evolution rests predominantly on pharmacological interrogation with Ompenaclid, a compound purported to exhibit selectivity for SLC6A8. The documented dose-dependent attenuation of SLC6A8 together with its downstream glycolytic mediators (HK2, ALDH1B1, PFKP) supports specific target engagement; yet we recognize that chemical inhibition per se cannot definitively rule out off-target liabilities or establish unequivocal genetic causation. Definitive confirmation will necessitate orthogonal genetic approaches—including siRNA/shRNA knockdown, CRISPR-Cas9-mediated deletion, and rescue paradigms—which constitute a priority in our ongoing research agenda. Third, leveraging TCGA as the discovery filter may have skewed gene selection toward biology characteristic of localized disease rather than systemic metastatic dissemination. It is noteworthy that neither TCGA nor GEO presently hosts dedicated mHSPC tumor tissue collections annotated with rigorously adjudicated mCRPC progression endpoints, a constraint that precluded confining the discovery phase exclusively to the target clinical stage. Fourth, both the single-cell transcriptomic analysis (GSE288878) and the functional characterization were restricted to the C4-2B cell line. Although this singular model preserves methodological continuity between biomarker identification and mechanistic elucidation, it circumscribes the applicability of our conclusions across the recognized molecular diversity of CRPC. C4-2B, being AR-positive and derived from bone metastases, reasonably approximates the mHSPC-to-mCRPC transition; nevertheless, CRPC comprises heterogeneous subtypes such as 22Rv1 (AR-positive, AR-V7-expressing), DU145, and PC3 (both AR-negative and androgen-independent), whereas LNCaP represents a hormone-sensitive rather than castration-resistant phenotype. These divergent dependencies on AR signaling and variable degrees of lineage plasticity may differentially impinge upon the oncogenic relevance and metabolic reliance on SLC6A8. Hence, our *in vitro* observations await corroboration across a broader panel of CRPC cell line models prior to any expansive therapeutic inference. Fifth, inter-individual heterogeneity in leukocyte subset proportions could have exerted confounding effects on the observed gene expression patterns. Regrettably, no scRNA-seq dataset of peripheral blood from treatment-naïve mHSPC patients is currently deposited in major archives (GEO, ArrayExpress, SRA), rendering cell-type deconvolution and proportional adjustment infeasible for the present cohort. We acknowledge this as a salient unresolved confounder and underscore that future scRNA-seq profiling in independent mHSPC series will be essential to pinpoint the cellular provenance of the prognostic signature. Sixth, the nomogram yields reliable projections only for the 1-year and 1.5-year intervals; estimation of 2-year or 3-year mCRPC-free survival probabilities is precluded by inadequate cumulative follow-up duration. Finally, the validation set was small in size, and the tally of mCRPC progression events was insufficient relative to model complexity. This sample-event imbalance engendered broad confidence intervals around performance indices, exemplified by the AUC (0.83; 95% CI, 0.62–1.00) and calibration metrics. Although point estimates suggested satisfactory model behavior, the statistical precision of these estimates was constrained by limited numbers. Furthermore, the risk cutoff was optimized within this restricted dataset and may warrant recalibration upon accrual of additional patients and events. Thus, the validation results merit prudent interpretation, and prospective confirmation in expanded, multicenter settings is obligatory before clinical implementation can be entertained.

## 5 Conclusion

The prognostic model demonstrated favorable predictive performance for mCRPC-free survival. SLC6A8 may function as a potential prognostic biomarker that accelerates prostate cancer progression, potentially by enhancing glycolytic metabolism.

## Data Availability

The data presented in the study are deposited in the Figshare repository, DOI: 10.6084/m9.figshare.34167933. Publicly available datasets analyzed in this study include GSE288878 (GEO, https://www.ncbi.nlm.nih.gov/geo/) and TCGA-PRAD (https://portal.gdc.cancer.gov/).

## Glossary

mHSPC: metastatic hormone-sensitive prostate cancer
mCRPC: metastatic castration-resistant prostate cancer
nmHSPC: non-metastatic hormone-sensitive prostate cancer
HSPC: hormone-sensitive prostate cancer
CRPC: castration-resistant prostate cancer
PRAD: prostate adenocarcinoma
ADT: androgen deprivation therapy
ARPIs: androgen receptor pathway inhibitors
AR-V7: androgen receptor splice variant 7
NHT: novel hormonal therapy
LHRH: luteinizing hormone-releasing hormone
PSA: prostate-specific antigen
PCWG3: Prostate Cancer Clinical Trials Working Group 3
ECOG: Eastern Cooperative Oncology Group
BMI: body mass index
OS: overall survival
PFS: progression-free survival
AUC-ROC: area under the receiver operating characteristic curve
CTCs: circulating tumor cells
scRNA-seq: single-cell RNA sequencing
DEGs: differential expressed genes
HVGs: highly variable genes
PCA: principal component analysis
t-SNE: t-distributed stochastic neighbor embedding
UMAP: Uniform Manifold Approximation and Projection
WGCNA: weighted gene co-expression network analysis
GO: Gene Ontology
KEGG: Kyoto Encyclopedia of Genes and Genomes
MF: molecular function
BP: biological process
CC: cellular component
TOM: topological overlap matrix
KME: module eigengene connectivity
LC-MS: liquid chromatography-mass spectrometry
ESI: electrospray ionization
OPLS-DA: orthogonal projections to latent structures discriminant analysis
PLS-DA: partial least squares discriminant analysis
PCA: principal component analysis
SHAP: SHapley Additive exPlanations
DCA: decision curve analysis
PPV: positive predictive value
NPV: negative predictive value
OOB: out-of-bag
SRM: Structural Risk Minimization
VC dimension: Vapnik-Chervonenkis dimension
SVM: support vector machine
LASSO: Least Absolute Shrinkage and Selection Operator
KNN: K-nearest neighbors
RF: Random Forest
AdaBoost: Adaptive Boosting
Elastic Net: Elastic Net regularization
GEO: Gene Expression Omnibus
TCGA: The Cancer Genome Atlas
GSE: GEO Series
glmnet: Generalized Linear Models with Elastic Net Regularization
Seurat: Seurat (single-cell analysis toolkit)
SingleR: SingleR (automated cell type annotation)
scMetabolism: scMetabolism (single-cell metabolic analysis)
LIMMA: Linear Models for Microarray Analysis
StringTie: StringTie (transcript assembly and quantification)
RSEM: RNA-Seq by Expectation-Maximization
HISAT2: Hierarchical Indexing for Spliced Alignment of Transcripts 2
fastp: fastp (ultrafast all-in-one FASTQ preprocessor)
clusterProfiler: clusterProfiler (universal enrichment tool)
rms: Regression Modeling Strategies
ImageJ: ImageJ (image processing and analysis)
LNCaP: Lymph Node Carcinoma of the Prostate
FGC: Fast Growing Colony
RIN: RNA integrity number
DV200: percentage of RNA fragments >200 nucleotides
RQN: RNA quality number
FPKM: fragments per kilobase of transcript per million mapped reads
TPM: transcripts per million
logFC: log fold change
min.pct: minimum percentage
CCK-8: Cell Counting Kit-8
FITC: fluorescein isothiocyanate
PI: propidium iodide
PVDF: polyvinylidene fluoride
SDS-PAGE: sodium dodecyl sulfate-polyacrylamide gel electrophoresis
RIPA: radioimmunoprecipitation assay
BCA: bicinchoninic acid
ECL: enhanced chemiluminescence
HRP: horseradish peroxidase
TBST: Tris-buffered saline with Tween-20
cDNA: complementary DNA
PE150: paired-end 150 bp sequencing
oligo(dT): oligo-deoxythymidine
PCR: polymerase chain reaction
SEM: standard error of the mean
IQR: interquartile range
VIP: variable importance in projection
ESI+: positive electrospray ionization mode
ESI-: negative electrospray ionization mode
MSn: tandem mass spectrometry
cps: counts per second
SLC6A8: solute carrier family 6 member 8
SLC25A39: solute carrier family 25 member 39
GRB10: growth factor receptor-bound protein 10
SELENBP1: selenium binding protein 1
YBX3: Y-box binding protein 3
HK2: hexokinase 2
PFKP: phosphofructokinase, platelet
ALDH1B1: aldehyde dehydrogenase 1 family member B1
AKT: protein kinase B
AMPK: AMP-activated protein kinase
GSH: glutathione
NAD^+^: nicotinamide adenine dinucleotide (oxidized)
NADH: nicotinamide adenine dinucleotide (reduced)
AUC: area under the curve
ROC: receiver operating characteristic
FDR: false discovery rate.
