# Supplementary material for: Peripheral blood mRNA-based prognostic model for progression to metastatic castration-resistant prostate cancer and identification of SLC6A8 as a glycolysis-promoting biomarker
Source: Front Oncol. 2026 Oct 14;16:1942820. doi: 10.3389/fonc.2026.1942820 (PMC13713402; doi:10.3389/fonc.2026.1942820)
Supplement: Supplementary file 1 [file DataSheet1.docx]

**Supplementary Figure1	Workflow of this study**

**Supplementary Table S1. TRIPOD checklist for reporting the development and internal validation of the prognostic model**

| **Section/Topic** | **Item** | **TRIPOD recommendation** | **Where reported in the manuscript** |
| --- | --- | --- | --- |
| Title and abstract | 1 | Identify the study as developing and/or validating a multivariable prediction model, the target population, and the outcome to be predicted. | Title; Abstract |
|  | 2 | Provide a structured summary of objectives, study design, setting, participants, sample size, predictors, outcome, statistical analysis, results, and conclusions. | Abstract |
| Introduction: Background and objectives | 3a | Explain the medical context and rationale for developing or validating the prediction model, including references to existing models. | Introduction, paragraphs 1-3 |
|  | 3b | Specify the objectives, including whether the study concerns model development, validation, or both. | Introduction, final paragraph |
| Methods: Source of data | 4a | Describe the study design or source of data separately for model development and validation. | Sections 2.3 and 2.3.6 |
|  | 4b | Specify the key study dates, including the beginning and end of recruitment and the end of follow-up. | Sections 2.3 and 2.3.3 |
| Methods: Participants | 5a | Specify the study setting, including the number and location of centers. | Section 2.3 |
|  | 5b | Describe the eligibility criteria for participants. | Section 2.3.1 |
|  | 5c | Describe the treatments received, if relevant. | Sections 2.3.1 and 2.3.3 |
| Methods: Outcome | 6a | Clearly define the outcome predicted by the model, including how and when it was assessed. | Section 2.3.3 |
|  | 6b | Report any actions taken to blind outcome assessment to predictor information. | Section 2.3.3 |
| Methods: Predictors | 7a | Clearly define all predictors used in model development and how and when they were measured. | Sections 2.3.3-2.3.6 |
|  | 7b | Report any actions taken to blind predictor assessment to outcome and other predictor information. | Standardized RNA sequencing and bioinformatic procedures were used; laboratory blinding status |
| Methods: Sample size | 8 | Explain how the study sample size was determined. | Section 2.3.2 |
| Methods: Missing data | 9 | Describe how missing data were handled and provide details of any imputation method. | Statistical Analysis section |
| Methods: Statistical analysis methods | 10a | Describe how predictors were handled in the analyses. | Sections 2.3.5 and 2.3.6 |
|  | 10b | Specify the model type, model-building procedures, and procedures used for predictor selection. | Section 2.3.6 |
|  | 10c | For validation, describe how predictions were calculated using the model developed in the development dataset. | Section 2.3.6 |
|  | 10d | Specify all measures used to assess model performance and, where relevant, compare multiple models. | Section 2.3.6 |
|  | 10e | Describe any model updating arising from validation, if performed. | Not applicable; no model updating was performed |
| Methods: Risk groups | 11 | Provide details on how risk groups were created, if applicable. | Section 2.3.6 |
| Methods: Development versus validation | 12 | For validation, identify any differences from the development data in setting, eligibility criteria, outcome, or predictors. | Not applicable to independent external validation; both sets were obtained by random splitting of the same prospective cohort |
| Results: Participants | 13a | Describe the flow of participants through the study, including the number with and without the outcome and the follow-up time. | Results; patient screening flowchart |
|  | 13b | Describe participant characteristics, including predictors and the number of participants with missing data. | Table 1 |
|  | 13c | For validation, compare the distribution of important variables with those in the development dataset. | Table 1 |
| Results: Model development | 14a | Specify the number of participants and outcome events included in each analysis. | Results; Table 1 |
|  | 14b | If performed, report the unadjusted association between each candidate predictor and the outcome. | Not available, because we used Elastic Net-regularized Cox model |
| Results: Model specification | 15a | Present the complete prediction model so that individual predictions can be made. | Results; model formula and nomogram |
|  | 15b | Explain how to use the prediction model. | Nomogram figure |
| Results: Model performance | 16 | Report performance measures with confidence intervals. | Results; time-dependent AUC, calibration curves, and decision-curve analysis |
| Results: Model updating | 17 | Report results from any model updating, if performed. | Not applicable |
| Discussion: Limitations | 18 | Discuss study limitations, including nonrepresentative samples, low event numbers, and potential overfitting. | Discussion, limitations paragraph |
| Discussion: Interpretation | 19a | For validation, discuss results with reference to performance in the development data and other relevant studies. | Discussion |
|  | 19b | Provide an overall interpretation considering objectives, limitations, results from similar studies, and other relevant evidence. | Discussion |
| Discussion: Implications | 20 | Discuss the potential clinical use of the model and implications for future research. | Discussion and Conclusion |
| Other information: Supplementary information | 21 | Provide information on the availability of the study protocol, web calculator, dataset, or other supplementary resources. | Data availability statement; Supplementary Materials |
| Other information: Funding | 22 | Report the sources of funding and the role of the funders. | Funding statement |

**Abbreviations:** TRIPOD, Transparent Reporting of a multivariable prediction model for Individual Prognosis Or Diagnosis; mHSPC, metastatic hormone-sensitive prostate cancer; mCRPC, metastatic castration-resistant prostate cancer; PCWG3, Prostate Cancer Clinical Trials Working Group 3; AUC, area under the receiver operating characteristic curve.

**Note:** The validation performed in this study was an internal validation based on a random split of the same prospective cohort; no independent external validation was performed. Replace bracketed author queries with final page and line numbers or confirmed methodological details before submission.
